# Effective delivery of social and behavior change communication through a Care Group model in a supplementary feeding program

**DOI:** 10.1186/s41043-017-0111-3

**Published:** 2017-09-12

**Authors:** Lauren Wilner, Devika J. Suri, Breanne K. Langlois, Shelley Marcus Walton, Beatrice Lorge Rogers

**Affiliations:** 1 Friedman School of Nutrition Science and Policy, Tufts University, 150 Harrison Ave, Boston, MA USA; 2 Division of Global Health Protection, Center for Disease Control and Prevention, Atlanta, GA USA; 3 Department of Nutritional Sciences, University of Wisconsin-Madison, Madison, WI USA

**Keywords:** Social and behavior change communication, Supplementary feeding, Moderate acute malnutrition, Food aid, Corn soy blend

## Abstract

**Background:**

In 2014, an intervention aimed at increasing the oil in corn soy blend (CSB) porridge prepared by caregivers of children with moderate acute malnutrition was implemented in Southern Malawi. This analysis describes the flow of key messages delivered through the Care Group model during this intervention.

**Methods:**

The intervention provided a supplementary food ration of CSB and oil and used a Care Group model in which healthcare workers were trained to deliver social and behavior change communication (SBCC) to care group volunteers who then delivered messages to caregivers of beneficiary children. Healthcare workers also delivered messages to caregivers directly. Interviews and focus groups were conducted with all three groups in order to determine the exchange of key messages about ingredient use, storage, and purpose, which were analyzed descriptively.

**Results:**

Analysis of SBCC flow and information exchange showed that 100% of caregivers reported learning about the amounts of oil and CSB to use while preparing porridge and over 90% of caregivers, healthcare workers, and care group volunteers reported talking about it. Focus groups confirmed an effective flow of communication among these three groups.

**Conclusion:**

This analysis evaluated the flow of key SBCC messages through multiple, overlapping lines of communication among healthcare workers, care group volunteers, and caregivers; the effective transmission of these SBCC messages through this model may contribute to the success of a supplementary feeding intervention program.

**Trial registration:**

The study was registered at ClinicalTrials.gov (NCT01873196).

**Electronic supplementary material:**

The online version of this article (10.1186/s41043-017-0111-3) contains supplementary material, which is available to authorized users.

## Background

Moderate acute malnutrition (MAM) affects over 50 million children worldwide, increasing their risk for poor development, disease, and death [1, 2]. Treatment of MAM frequently relies on distribution of nutrient-dense supplementary foods, such as corn soy blend (CSB), to households with affected children. Nutritional improvements to CSB have recently been recommended, including the addition of a fortified oil ration, which contains added micronutrients and improves the energy density of the prepared porridge when added at the target ratio of 30 g oil to 100 g CSB [3]. Thus, effectiveness of these food products for treatment of malnutrition depends on compliance with storage, use, and preparation instructions. We previously conducted a feasibility and acceptability study among caregivers of children in a MAM treatment program in Malawi and found that providing social and behavior change communication (SBCC) to encourage adequate use of oil in prepared porridge (in accordance with the aforementioned ratio), along with a ration of CSB and oil in sufficient quantity to permit the target ratio, increased the amount of added oil in CSB porridge prepared by caregivers of beneficiary children [4]. These messages were part of a suite of messages aimed at ensuring that the intended nutritional benefits of the supplementary feeding program reached the target child.

Many studies show that intensive, targeted SBCC messaging—evidence-based communication strategies to promote behaviors that improve health outcomes—can enhance the effectiveness of nutritional interventions [5–7]. A study by Roy et al. (2005) [5] found that groups receiving intensive nutrition education, some with supplementary foods and some without, all experienced increased weight gain and improved nutritional status over the course of the study period. A study in Thailand found that health education messages can improve various health outcomes, including nutritional status, whether the subjects received supplementary food or not [8]. Other studies have similarly found efficacy in nutritional education in improving health status of children.

The intervention discussed in this paper provided health communication on site at the food distribution point through healthcare workers in the community and reinforced the messages using the “Care Group” model in order to ensure effective communication of the SBCC messages to caregivers. A care group is typically 10–15 volunteer, community-based health educators (“care group volunteers” or CGVs) who regularly meet together with project staff for training and supervision. They are different from typical mother’s groups in that each volunteer is responsible for regularly visiting and holding group meetings with 10–15 of her neighbors, sharing what she has learned, and facilitating behavior change at the household level. Care groups create a multiplying effect to equitably reach every beneficiary household with interpersonal behavior change communication. They also provide the structure for a community health information system that reports on new pregnancies, births, and deaths detected during home visits [9]. The Care Group model is currently growing in popularity as an effective approach to maternal and child health interventions, including nutritional interventions [10, 11]. A review of 19 USAID-funded NGO Child Survival and Health Grants Programs—10 of which used the Care Group model and 9 of which did not—found that those using the Care Group model had double the coverage of non-care group programs [12].

The Care Group model and SBCC are common elements included in supplementary food aid distribution. However, the specific effects of their inclusion in the communication flow with which they are implemented remain unknown. The combined intervention of additional oil with SBCC provided through multiple channels was effective in achieving an increased ratio of oil to CSB in caregiver porridge preparation [4]. The goal of this intervention was to improve the nutritional content of the supplementary food reaching beneficiary children via proper preparation. This paper describes the elements of the SBCC that contributed to its effectiveness: (1) the components of the SBCC messages and (2) the communication flow through the healthcare system and the Care Group model.

## Methods

### Study design and setting

**Fig. 1:**
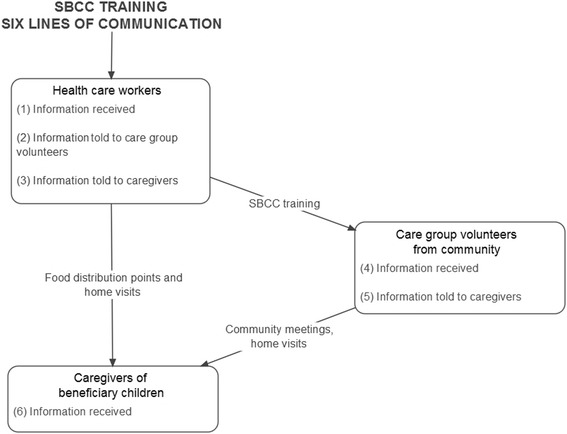
Flow of SBCC messages in the care group model used in this study

This is a descriptive analysis of data from a cross-sectional study examining whether caregivers of beneficiary children 6–59 months of age enrolled in a supplementary feeding program for treatment of MAM would prepare and feed CSB porridge as directed given enough oil and specific SBCC messaging. The methods and main results of this study are published elsewhere [4]. Briefly, the study was conducted among caregivers of beneficiaries from a USAID, Office of Food for Peace supplementary feeding program for children with MAM implemented by Catholic Relief Services called Wellness and Agriculture for Life Advancement (WALA) in four districts in Southern Malawi from March to July 2014. The study consisted of three groups. The control group received the standard WALA food ration of 8 kg of CSB in bulk and 1 L of fortified vegetable oil per month for four consecutive months and standard SBCC covering general health messages on family planning, HIV, vaccinations, and other topics. Intervention group 1 received the same ration of CSB but an increased oil ration of 2.6 L and intensified SBCC emphasizing the target oil:CSB ratio. Intervention group 2 received the same ration and SBCC as group 1, but the CSB ration was distributed as four 2-kg bags with printed behavior change and preparation messaging on the package (Table 1). The WALA program used the Care Group model as part of its intervention. Using the Care Group model for the dissemination of SBCC in this study, experts trained supervisory healthcare workers in key SBCC messages; these healthcare workers then trained care group volunteers who are from the community. Each care group volunteer was responsible for providing communication about key SBCC messages to approximately 10 caregivers through home visits and community meetings. The healthcare workers who trained the care group volunteers also communicated directly with caregivers at food distribution points and through occasional home visits (Fig. 1).

**Table 1:** Description of study groups

	Intervention group 1	Intervention group 2	Control group
Supplementary food (distributed monthly for the period of 4 months)	8 kg CSB (bulk) + 2.6 L fortified vegetable oil	8 kg CSB (in 4 2-kg packages) + 2.6 L fortified vegetable oil	Standard program ration: 8 kg CSB (bulk) + 1 L fortified vegetable oil
SBCC	Intensified SBCC emphasizing the recommended preparation method, oil to CSB ratio, and targeting of supplement to beneficiary child	Intensified SBCC emphasizing the recommended preparation method, oil to CSB ratio, and targeting of supplement to beneficiary child; the repackaged CSB also included printed SBCC messaging	Standard program SBCC: general health messages on family planning, HIV, vaccinations, and other topics

The SBCC in this study was specifically formulated by an SBCC team including Pakachere Institute of Health and Development Communication in Malawi, experts from the WALA program, and the Tufts University research coordinator [13]. The SBCC component formulated aimed “to develop relevant SBCC messages that would be disseminated through... training modules for the community [healthcare workers] and pamphlets for [care group volunteers], as well as banners to be displayed at the food distribution points and back-of-packet messaging... to promote compliance among [caregivers]” [13]. Formative research and piloting of these materials was performed using focus group discussions, recipe-testing trial sessions, a message design workshop, and a creative workshop. Additionally, training materials for healthcare workers and care group volunteers were distributed, and food distribution point banners were designed. This formative research identified four main behavior areas that should be the key components in the SBCC intervention: “Measurement of ingredients when preparing CSB porridge with oil; length of CSB porridge cooking time; use of oil for purposes other than its intended use in CSB porridge preparation; and sharing of CSB porridge with other family members” [13]. An example of materials developed can be seen in Additional file 1: Figure S1.

### Participants

The original sample size of 200 caregivers of beneficiary children per group was based on the detection of differences among the study groups for the primary outcomes of mean oil to CSB ratio in porridge prepared by caregivers and proportion of caregivers reaching or exceeding the target ratio [4]. Food distribution points were allocated to study groups purposively in order to ensure that those receiving a program change (intervention groups) and those receiving no program change (control) were geographically separate from each other to avoid potential spill-over or contamination. Caregivers of beneficiary children were then randomly selected, based on their attendance at the food distribution point that day, to participate in an in-home interview, which included questions on enrollment and experience in the WALA treatment program, who consumed the ration, preparation of CSB porridge, and handling and storage of rations, as well as their exposure to SBCC messages through the various channels. Healthcare workers and care group volunteers were selected to participate in interviews to assess their knowledge of the SBCC messages, from whom they learned, and what they were communicating to the caregivers.

### Quantitative data collection and analysis

The two intervention groups received the same SBCC, and did not differ significantly with respect to the primary outcome of the study, oil to CSB ratio [4]; thus for the purpose of this analysis, we analyze the two intervention groups together. We do not include the control group in this analysis, as they did not receive the intensified SBCC intervention regarding CSB preparation, handling, and feeding practices, which are the subject of this analysis. Data were analyzed descriptively—no hypothesis testing was conducted.

Quantitative surveys with caregivers, healthcare workers, and care group volunteers all contained questions regarding information they received, from whom, and information they provided, and to whom. The purpose of this paper is to report the flow of communication and information exchange. There were six lines of communication analyzed: (1) information the healthcare workers reported receiving; (2) information the healthcare workers reported providing the care group volunteers; (3) information the healthcare workers reported providing the caregiver; (4) information the care group volunteers reported receiving; (5) information the care group volunteers reported providing the caregiver; (6) information the caregiver reported receiving. This exchange of information analyzed came from responses to a series of yes/no questions in the questionnaires, formulated as “did they tell you about….?” or “did you tell them about…?” related to five SBCC messages: ingredients to use in porridge preparation, who to feed the porridge to, how often to feed porridge to the beneficiary child, how to store the CSB, and how to store the oil (Table 2). For each of the six lines of communication, the frequency of responses on each of the five SBCC messages were tabulated, and radial graphs were made for main points of communication. In addition, frequencies of caregivers’ reported home visits from healthcare workers and care group volunteers were tabulated. Quantitative data analysis was performed using Stata 13.1 and Microsoft Excel.

**Table 2:** SBCC questions^1^ included on in-depth interviews to evaluate communication reportedly received and given^2^ among healthcare workers, care group volunteers, and caregivers

	Healthcare workers	Care group volunteers	Caregivers
SBCC messages received	(1) From training	(4) From HCW	(6) From HCW or CGV
	1. Did they^3^ tell you about the ingredients that should be used to prepare CSB porridge? 2. Did they tell you how often to feed the porridge to the child? 3. Did they tell you who should eat the porridge? 4. Did they tell you how to store the CSB? 5. Did they tell you how to store the oil?		
SBCC messages given	(2) To CGVs and (3) caregivers	(5) To caregivers	N/A
	1. Do you tell them about the ingredients to use when preparing CSB porridge? 2. Do you tell them on how often to feed the child? 3. Do you tell them on who to feed the porridge to? 4. Do you tell them how to store the CSB? 5. Do you tell them how to store the oil?		

*Abbreviations*: *HCW* healthcare worker, *CGV* care group volunteer, *SBCC* social and behavior change communication, *CSB* corn soy blend, *N/A* not applicable

^1^Response options were yes, no, or cannot remember/do not know. Healthcare workers and CGVs were both asked about what they were taught during training and what they taught to caregivers

^2^The six channels of communication evaluated are (1) information the HCW reported receiving; (2) information the HCW reported telling the CGV; (3) information the HCW reported telling the caregiver; (4) information the CGV reported receiving; (5) information the CGV reported telling the caregiver; (6) information the caregiver reported receiving

^3^“They” and “them” refer to the group that is either giving or receiving the SBCC messages. For example, “they” in the case of HCWs would be their trainers, in the case of CGVs would be the HCWs, and in the case of caregivers could be either the CGV or HCW

### Qualitative data collection and analysis

Semi-structured focus group discussions were conducted among caregivers of beneficiary children and care group volunteers to gather information about the SBCC intervention that might not have been included in the quantitative questionnaires. For caregivers, focus group discussions gathered content about: porridge preparation (including how they learn); use of the ration (including sharing, diversion to cooking of other dishes, and selling); porridge consumption; feelings and perceptions of the porridge; and experiences with the supplementary feeding program. For care group volunteers, discussions included the following: roles within the community; what they tell caregivers; and interaction with caregivers.

Focus group discussions were recorded, transcribed, and imported into QSR International’s NVivo 10 software for analysis. Broad categories were discussed and agreed upon by two analysts after each independently reviewed the transcripts. Data were categorized according to the broad structural domains, and then an inductive approach was used to identify common conceptual themes through line-by-line analysis of the text. Themes related to the flow of communication and knowledge between care group volunteers and caregivers were identified.

## Results

### Quantitative

**Fig. 2:**
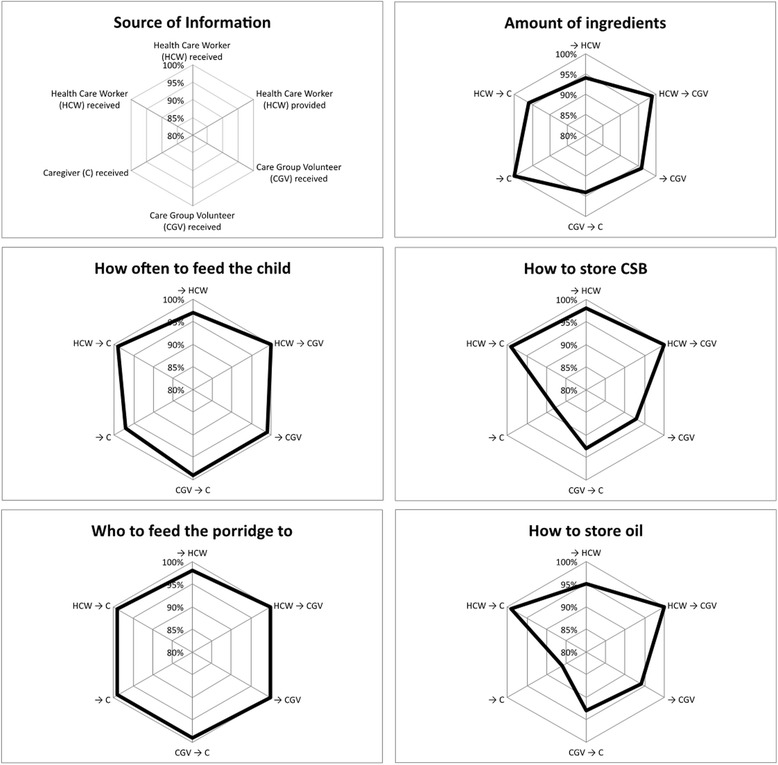
Percent reporting they provided or received each of five key SBCC messages during the program. The arrows indicate direction of information flow: at each point of each graph, those on the left side of the arrow are providing information and those on the right side of the arrow are receiving information

Interviews from 388 caregivers, 135 healthcare workers and 206 care group volunteers were analyzed. Overall, reported communication of the five SBCC messages was above 80% among all six channels (Fig. 2). The SBCC message on the amount of ingredients to use was reported to be received by 99% of caregivers; while healthcare workers and care group volunteers reported lower rates of communicating this topic with caregivers, the overlapping lines of communication may have resulted in the caregivers’ level of reported receipt being so high. The SBCC topics of who to feed the porridge to and how often were most consistently reported as being provided or received by all groups, while storage of CSB and oil were the least reported as being received by caregivers. Since enrollment, only 25% of caregivers reported having a home visit; of these visits, 57% reported receiving them from care group volunteers, and 100% reported receiving these visits from healthcare workers. However, 90% of caregivers reported first learning about porridge preparation at the food distribution point, and only 2% of caregivers reported first learning from the healthcare workers, indicating the prominence of learning from the care group volunteers within the communities.

Figure 2 shows the content of the information exchanged during these instances of communication and the reported reception and provision of communication among the caregivers, healthcare workers, and care group volunteers. At every line of communication analyzed, at least 85% of respondents reported reception or provision of the five SBCC messages. As the primary objective of this study was to increase the ratio of oil to CSB in caretaker-prepared porridge, amount of ingredients was a key SBCC message, and 100% of caregivers reported being told about the amount of ingredients to use: about 90% from the healthcare workers, and about 90% from the care group volunteers, so that between these two sources of information, 100% of caregivers received the message (and clearly, with significant overlap between the two sources). Over 90% of care group volunteers and healthcare workers reported receiving information on this topic, indicating that there was exchange of information on this key message. Over 95% of all reports indicated information exchange on the topic of frequency of feeding, as well as on the topic of to whom the porridge should be fed. Over 95% of healthcare workers reported receiving education and providing education to care group volunteers and to caregivers regarding storage of ingredients, but the messages about storage appeared to be least well received by caregivers, with under 85% of caregivers reporting that they received these messages.

### Qualitative

Nine focus group discussions were conducted among caregivers of beneficiary children and nine were conducted among care group volunteers. Each focus group contained between 6 to 10 participants and lasted for 1 h and 24 min, on average. Several themes related to the flow of communication and knowledge between care group volunteers and caregivers were identified. Example quotes are displayed in Table 3.

**Table 3:** Example quotes from care group volunteers and caregivers

Theme	Participant	Example quote
Roles within the community	Care group volunteers	“we help in identifying malnourished children and referring them to PCI health workers”
		“we also go into the village to find beneficiaries”
		“we meet once every month with the health promoter and they teach us how we can work in our areas”
		“we go to the households of beneficiary children to see how they prepare the ration and we check if they are following the instructions”
		“We inspect homes of beneficiary children to see if the mothers are properly feeding the children; if they are not, we remind them of the instructions that they were given.”
		“We also empower women not to ultimately rely on the ration that they get … through teaching them how to prepare nutritious food from locally available materials.”
Communication process	Care group volunteers	“The health promoter comes to teach us on cooking porridge and other foods. Finally, we meet at one household where we teach the women what the health promoters taught us.”
		**“**They teach us different things which in turn we deliver to the women.”
	Caregivers	“Yes, we do receive instructions on how to cook and take care of the ration. ”
		“The Health Promoters taught us all this.”
Knowledge exchange	Care group volunteers	“We teach the women how to prepare and feed the child corn soy blend porridge”
		“we tell them that they should practice hygiene and the household should be clean”
		“I encourage women not to sell or share the ration”
	Caregivers	“Yes, we were told to first of all clean a pot and measure corn soy blend into the pot, then add 6 tablespoons of oil into the corn soy blend; then add water and stir thoroughly. After that, we were told to put the pot on fire and cook the porridge for 40 min.”
		“We were told not to sell or share the ration”
		“We were told to take care of it and keep it safe from rats, roaches and dust”
		“they also tell us not to share the foods to neighbors because they are medicine to the children”
		“We were told to always use ration oil when preparing the porridge”
		“They teach us on measurements; how much of the CSB and oil to use. They also instruct us to cook the CSB porridge for the beneficiary child only and always to measure the recommended amount.”

Care group volunteers commonly described the flow of communication, how they learn from healthcare workers and subsequently teach caregivers what they learn. They described having positive roles within the community, which included looking after pregnant women and children, encouraging women to attend growth monitoring sessions, making referrals to the hospital or supplementary feeding program, teaching about proper sanitation and hygiene, visiting households, monitoring growth of children, encouraging breastfeeding, giving general guidance on care of pregnant women and mothers, and child feeding. Within the supplementary feeding program, specifically, care group volunteers described that they identified beneficiaries, worked with healthcare workers, taught about proper use and preparation of the food ration, visited beneficiary households, monitored compliance, encouraged caregivers to follow instructions, and advised them not to sell or share. Additionally, care group volunteers described that they advised caregivers about proper sanitation and hygiene, child feeding, and encouraged caregivers to use other foods in addition to the supplementary food ration.

Care group volunteers also recounted several important messages they communicated to caregivers relating to preparation and consumption of the porridge, sharing and selling, storage of the food, use of other foods, and the importance of the food ration. On their part, caregivers commonly described that they received instructions and guidance on how to prepare the porridge. Most commonly, caregivers not only described receiving instructions from healthcare workers but also described being taught how to prepare the porridge from care group volunteers, “people from Africare” [one of the implementing organizations], “people at the food distribution point,” and “other healthcare workers.” With regard to the messages they received, caregivers described being told how to properly prepare the porridge, proper storage, and cleanliness; that the ration is like medicine for the child; not to share or sell; to use correct measurements; not to give to husbands; to prepare for the beneficiary child only; to prepare the porridge always with oil; and to prepare the porridge three times a day.

## Discussion

This analysis found that, overall, the reported receipt and provision of SBCC messages was very high in this program, which used the Care Group model to communicate and reinforce pre-formulated SBCC messages that were also provided by the healthcare workers. In reporting on provision of information, and the “delivery” side of the information exchange, this analysis found that the healthcare workers are providing information to both the care group volunteers and the caregivers, and likewise the care group volunteers are providing information to the caregivers. In addition, this analysis found that when on the “receiving” side of the SBCC exchange, a high percentage of all three groups (health care workers, care group volunteers, and caregivers) report overall reception of the messages, and that the messages most central to the intervention—ingredients to use, to whom to feed the porridge, and how frequently—were most consistently communicated and received by the caregivers.

We also found that the presence of multiple lines of communication were essential in this intervention. As intended in the model, most initial information exchange (90%) occurred at the food distribution point. However, about a quarter of caregivers reported home visits by the healthcare workers and care group volunteers, indicating the existence of a secondary point of communication. The Care Group model is based upon information exchange at various points of contact, and our results indicate this Care Group model SBCC intervention achieved this. Individual components of the SBCC were communicated at multiple places, from multiple people, and at multiple times. Additionally, we found consistency in what one group reported providing to another, and what the other group reported receiving. The care group volunteers reported telling the women about the amount of ingredients to use, and likewise the caregivers reported being told this; and this was largely true for each of the six communication lines discussed. For example, approximately 95% of healthcare workers and 95% of care group volunteers report educating caregivers on the amount of ingredients to use in porridge preparation; given that we find that 100% of caregivers report receiving this information, we can infer that having overlapping lines of communication was key in the successful SBCC coverage in this intervention. Additional evidence from the qualitative analysis also indicates that the SBCC intervention was successful, and the knowledge was received by caregivers.

The primary study results showed that caregivers who received the intervention (additional food ration and intensive SBCC) were more likely to feed the porridge exclusively to the beneficiary child and were more likely to attain the target ratio in porridge preparation compared with caregivers in the control group given standard programming [4]. In combination with this, our results showing effective SBCC communication within the intervention groups may indicate that the SBCC strategy contributed to the success of the overall intervention. A study by George et al. (2015) found similar effectiveness in delivery of SBCC through a Care Group model, as they found that intensive SBCC from care group volunteers, measured through care group volunteers visits to caregivers, positively impacted nutrition-related behaviors [11]. We believe that the SBCC component of our study, which we have found to be effectively implemented, is likely to have contributed, at least in conjunction with the increased oil ration, to the effectiveness of this intervention. While this study and aforementioned studies have shown that SBCC through the Care Group model is effective, there are few studies to evaluate the actual flow of the SBCC information among the healthcare workers, care group volunteers, and caregivers [11]. Through this analysis, we have displayed this flow and found that through multiple, overlapping lines of communication among multiple groups of people, we can effectively deliver SBCC and thereby see the success of a supplementary feeding program intervention.

This analysis has some limitations. One limitation is that the SBCC was always provided with supplementary food (including an increased oil ration), so it is not possible to distinguish between the effects of these two components. Another limitation is that the data in this study were self-reported, which may be vulnerable to the social desirability bias. Respondents may have indicated that they received or gave information as they were supposed to, rather than to reflect what they actually did [14]. Lastly, while we assessed whether communication on five different SBCC messages occurred, we did not evaluate the content of this communication in terms of the “correctness” compared to target SBCC messages. In order to discern the independent effects of all components of SBCC, future studies could examine interventions with and without intensive SBCC and also evaluate the accuracy of the communication itself.

## Conclusion

This study succeeded in delivering intervention-specific SBCC messages effectively through a Care Group model consisting of a combination of communication channels including healthcare workers, care group volunteers, and caregivers of program beneficiaries. The Care Group model was a key component of a larger strategy for SBCC, which included information exchange through the traditional care group lines of communication as well as through communication through healthcare workers and through the staff providing the ration at the food distribution point. In this analysis, we showed that individual components of the overall SBCC message were communicated at different points, between different people, and through different means—contributing to an overall high rate of reported communication of the SBCC messages.

## Additional file

Additional file 1: Pamphlet cover page: “Your child’s nutrients (health) in CSB-Oil porridge.” (DOCX 2746 kb)

## Abbreviations

CGV: Care group volunteer
CSB: Corn soy blend
HCW: Healthcare worker
MAM: Moderate acute malnutrition
SBCC: Social and behavior change communication
USAID: United States Agency for International Development
USDA: United States Department of Agriculture
WALA: Wellness Agriculture for Life Advancement
