# Supplementary figures and images for: Effective delivery of social and behavior change communication through a Care Group model in a supplementary feeding program
Source: J Health Popul Nutr. 2017 Sep 12;36:34. doi: 10.1186/s41043-017-0111-3 (PMC5596483; doi:10.1186/s41043-017-0111-3)

**Pamphlet Cover Page: “Your child’s nutrients (health) in CSB-Oil porridge”**


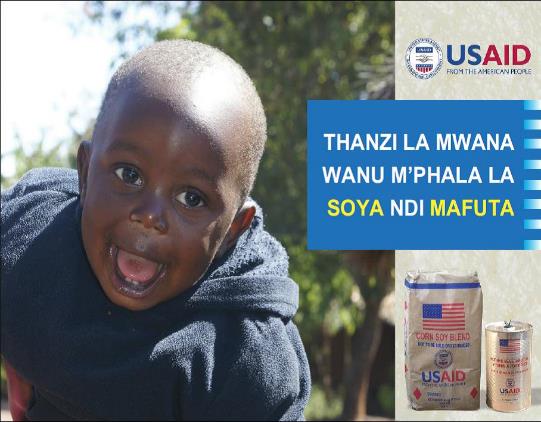


**Banner**


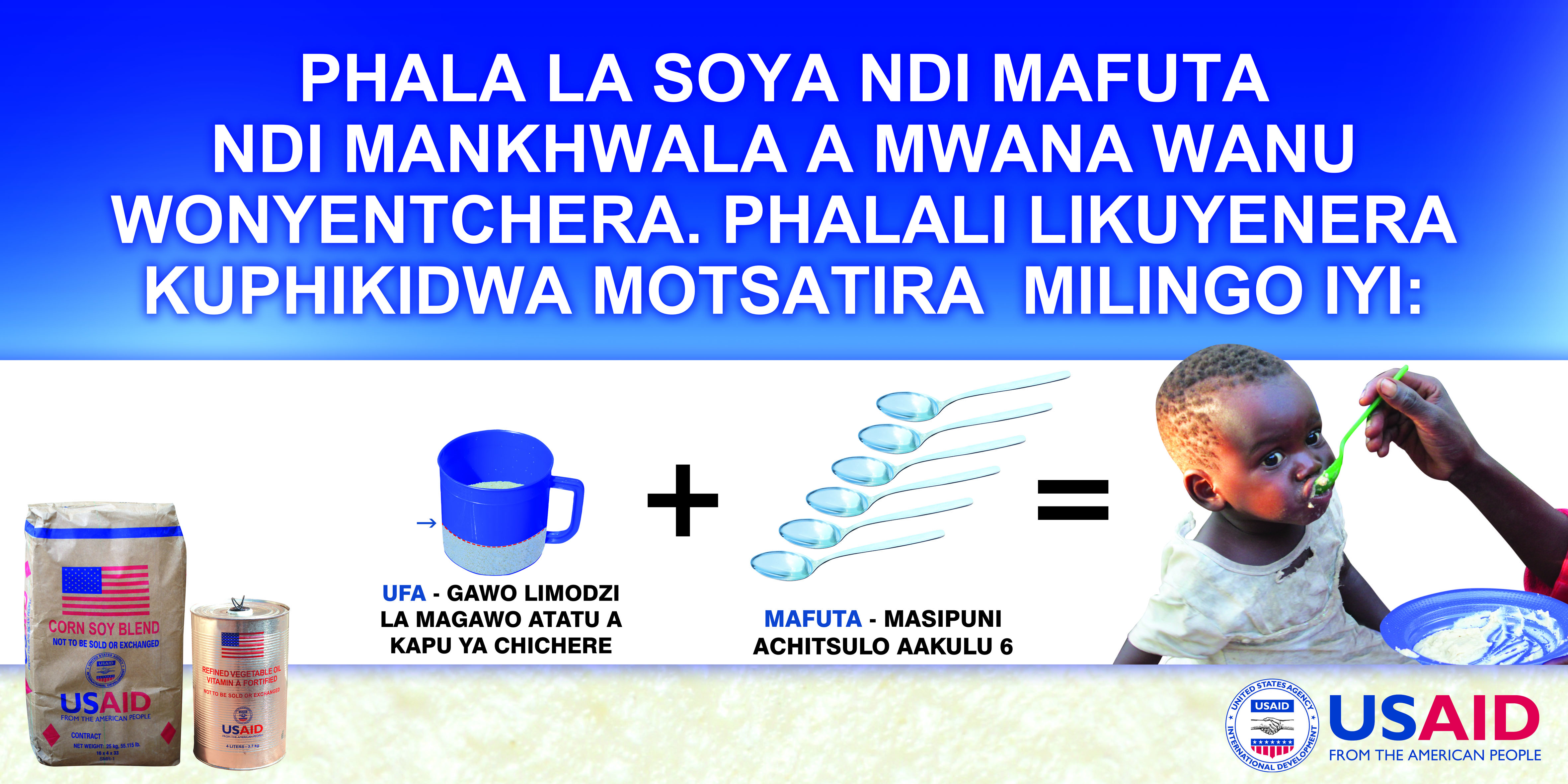


**Two kg CSB packets**


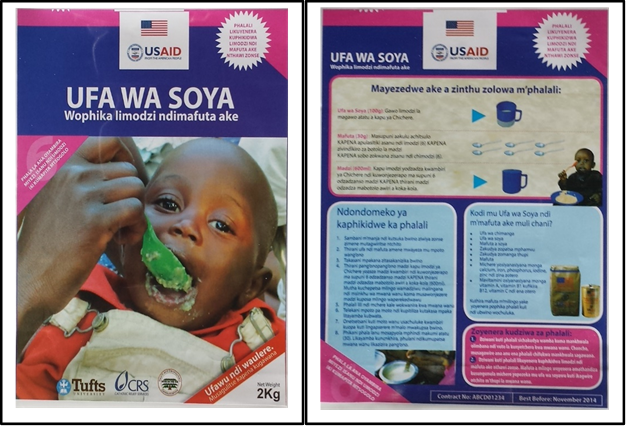

Supplement: Additional file 1: — Pamphlet cover page: “Your child’s nutrients (health) in CSB-Oil porridge.” (DOCX 2746 kb) [file 41043_2017_111_MOESM1_ESM.docx]
